# Electroacupuncture Ameliorates Cerebral Ischemic Injury by Inhibiting Ferroptosis

**DOI:** 10.3389/fneur.2021.619043

**Published:** 2021-03-08

**Authors:** Guangda Li, Xiaoxiao Li, Jianjian Dong, Yongsheng Han

**Affiliations:** ^1^Graduate School, Anhui University of Chinese Medicine, Hefei, China; ^2^Institute of Neurology, Anhui University of Chinese Medicine, Hefei, China

**Keywords:** electroacupuncture, ischemic stroke, ferroptosis, mitochondrial, iron homeostasis

## Abstract

**Background:** Our previous study found that electroacupuncture (EA) can promote the recovery of neurological functions, reduce the volume of cerebral infarction, and protect the neurovascular unit in middle cerebral artery occlusion (MCAO) rats. Some studies have shown that ferroptosis is closely related to ischemic stroke; however, whether EA plays a protective role by regulating ferroptosis is unknown.

**Objective:** We aimed to investigate the inhibitory effects of EA on ferroptosis in MCAO rats.

**Methods:** We used 36 adult male Sprague–Dawley rats in this study. MCAO rats were established according to the Zea method and treated with EA at a continuous wave of 2/100 Hz and ~2–4 V for 30 min for 7 consecutive days. We analyzed the coordinated motor deficit and volume of cerebral infarction *in vivo* through 9.4-tesla magnetic resonance imaging. Then, the ischemic brain tissue was isolated and the levels of malondialdehyde (MDA), superoxide dismutase (SOD), glutathione (GSH), and iron were determined. Western blotting and real-time quantitative PCR were performed to evaluate the expression of glutathione peroxidase 4 (GPX4), transferrin (Tf), transferrin receptor 1 (TfR1), and ferritin heavy chain 1 (FTH1). To confirm the results, we used a transmission electron microscope to observe the mitochondrial morphology.

**Results:** EA intervention significantly decreased the oxidative stress level and inhibited ferroptosis. EA significantly improved coordinated motor deficit (*P* < 0.01) and decreased cerebral infarct volume (*P* < 0.01) in the EA + MCAO group, compared with the MCAO group. EA downregulated the level of MDA (*P* < 0.01) and total iron (*P* < 0.01) and upregulated the level of SOD (*P* < 0.01) and GSH (*P* < 0.01) in the EA + MCAO group, compared with the MCAO group. EA increased the levels of GPX4 and GPX4 mRNA (*P* < 0.01) and FTH1 and FTH1 mRNA (*P* < 0.05, *P* < 0.01), whereas it decreased the levels of Tf and Tf mRNA (*P* < 0.05, *P* < 0.01) and TfR1 and TfR1 mRNA (*P* < 0.01) in the EA + MCAO group, compared with the MCAO group. EA also promoted the recovery of mitochondrial morphology according to the mitochondrial classification system for the ischemic cerebral tissue.

**Conclusion:** Our results indicate that EA can inhibit ferroptosis by regulating oxidative stress and iron-related proteins, thus conferring protection against MCAO in a rat model.

## Introduction

Stroke causes neuronal cell death and neurological dysfunction, and nearly 70% stroke cases are caused by cerebral ischemia (1). Ischemic cerebral stroke (ICS) is associated with high rates of morbidity, disability, and mortality, which cause serious economic burden to families and society at large. Recent studies have reported that ICS is characterized by oxidative stress (2), excitotoxic injury (3), inflammation (4), autophagy (5), and apoptosis (6). Ferroptosis is a unique form of cell death that significantly differs from classical apoptosis, necrosis, pyroptosis, or autophagy, and it is characterized by iron-dependent oxidative damage to membrane phospholipids involved in important pathological mechanisms of neurodegenerative disease (7). Studies have indicated that ferroptosis occurs in ICS, the levels of iron in the brain increased, and increasing iron export or applying the ferroptosis inhibitor significantly reduces the cerebral infarction and improve the neurological function score (8). Therefore, therapeutic targeting of ferroptosis could be a novel strategy for treating ICS in humans.

Electroacupuncture (EA) combines traditional acupuncture and modern electrical stimulation and has been widely used in the clinical setting. It can considerably improve the neurological function and quality of life of patients (9, 10). EA not only offers strong and stable stimulation but also can be applied to multiple targets (11, 12). Recent studies have shown that EA exhibits anti-apoptotic effects (13), inhibits autophagy (14), exhibits anti-inflammatory properties (15), reduces antioxidant stress (16), and promotes the proliferation and differentiation of neural stem cells (17), and neurovascular unit (NVU) remodeling (18). However, the mechanisms of action of EA in ischemic stroke are yet to be determined. The rat model of middle cerebral artery occlusion (MCAO) closely resembles human ICS, in which the middle cerebral artery (MCA), and its branches are often affected by different impactors (19). In this study, we attempted to determine the ferroptosis-inhibiting effects of EA in MCAO rats.

## Materials and Methods

### MCAO Model

An MCAO model was established to represent ischemic cerebral injury according to the Longa method (20). Briefly, rats were first anesthetized by intraperitoneally injecting 3% pentobarbital sodium (30 mg/kg). Then, a 3.0-cm monofilament nylon suture (L3200, Guangzhou Jialing Biotechnology Co., Ltd.) with a rounded tip coated with silicone rubber was inserted into the internal carotid artery (ICA) (18–20 mm) to block the MCA blood flow. The model was evaluated according to the following criteria: 0, normal limb activity; 1, difficulty stretching the right forelimb; 2, difficulty stretching the right forelimb along with a significant decrease in anti-lateral push ability; 3, the right forelimb was flexion and turned to the right as the animal crawled; and 4, inability to walk spontaneously or lack of self-consciousness. The model rats with three points were included in the study. These rats were able to move around in the cage but were unable to approach all the sides. The right forelimb moved minimally and turned to the right when the animal crawled, and it either reacted slowly or did not respond to stimulus on the right side.

### Grouping of Rats

Adult male Sprague–Dawley rats (*n* = 36) weighing 280 ± 20 g were provided by Jinan Pengyue Experimental Animal breeding Co., Ltd (No. SCXK2019-0003) (Jinan, China). The animals were housed in a quiet room under a controlled environment with 21–26°C temperature and 50–70% humidity. All experimental procedures were approved by the Animal Ethics Committees of Anhui University of Chinese Medicine and were conducted according to the guidelines provided by the Chinese Council on Animal Care.

All the 36 rats were randomly numbered and then divided into the following groups (*n* = 12/group): (i) Control group, in which the rats only underwent neck incision and exposure to the ICA; (ii) MCAO group, in which the left ICA was inserted with a nylon suture (18–20 mm); and (iii) EA +MCAO group, in which the surgical method was the same as that in the MCAO group. The balance supplement method was adopted to ensure the number of experimental cases in each group. The rats were treated with 30-min EA for 7 consecutive days 6 h after surgery. On day 7 after surgery, the rats were anesthetized by intraperitoneally injecting 3% pentobarbital sodium and were sacrificed to collect the samples.

### EA Treatment

We adopted an acupuncture acupoint map for rats developed by Li et al. (21). Briefly, 13-mm-long and 0.18-mm-wide needles (Wujiang Yunlong Medical equipment Co., Ltd., Wujiang, China) were transversely inserted into Baihui (GV20), Shuigou (GV26), bilateral Sanyinjiao (SP6), and bilateral Neiguan (PC6). Thereafter, a continuous wave of 2/100 Hz and ~2–4 V, with electrical stimulation, was applied for 30 min for 7 consecutive days. During the EA treatment, one rat died of weak breathing, which may be due to decrease in vital signs after the surgery and could be attributed to intolerance to the EA treatment.

### Coordinated Motion Experiments

We performed a coordinated movement test 7 days after inducing MCAO. Briefly, the rats were placed in coordinated motion detectors (YLS-30A, Jinan Yiyan Technology Development Co., Ltd.) designed for rodents and tested at the rate of 12 r/min. Initially, they were placed horizontally for ~1 min for adaptation, and then they were moved at an inclination of 60° from the ground. The motion time of the rats was recorded until they fell out of the roller.

### 9.4T MR Imaging

To determine the effect of EA on the cerebral infarction volume in MCAO rats, we used a 9.4-T/400-mm ultra-high field magnetic resonance (9.4-T MRI) system. Briefly, the rats were anesthetized with 3.5% isoflurane and oxygen and were laid flat on a rodent bed overlaid with a water bath mat for keeping them warm. A respiratory monitoring system was used to monitor vital signs, with their respiratory rate maintained at 30 ± 5 times/min. Image scanning was performed using the Agilent technology 9.4T/400 mm animal scanner (Agilent Technologies, Santa Clara, CA, USA), and T2-weighted images were captured using relaxation enhancement (RARE) sequences targeting the following parameters: repetition time (TR) = 6,000 ms, echo spacing (ESP) = 10,000 ms, field of view (FOV) = 23 × 23 mm^2^, excitation angle = 90°, refocusing angle = 180°, bandwidth = 40 kHz, echo train length (ETL) = 8, k-zero = 3, effective echo time (TE) = 30 ms, averages = 5, slices = 50, and thickness = 0.5 mm. Image-pro plus software was used to determine the cerebral infarction volume by using the following formula:

$$\begin{array}{l} \text{Infarct volumes}\left(\text{\%}\right)={\text{V}}_{\text{L}}/Vw\times \;100\% \end{array}$$

where V_L_ (mm^3^) = scanned lesioned area (mm^2^) × scanned relative thickness (mm); and

V_W_ (mm^3^) = scanned whole area (mm^2^) × scanned relative thickness (mm); V_L_ denotes scanned volumes of the lesioned brain issue, and V_W_ denotes scanned volumes of the whole brain issue.

### Determination of Malondialdehyde, Glutathione, Superoxide Dismutase, and Iron Levels

To confirm the effect of EA on ferroptosis regulation, the levels of malondialdehyde (MDA), glutathione (GSH), superoxide dismutase (SOD), and iron were determined. Briefly, the brain tissues were isolated and perfused with 0.9% sodium chloride solution. Then, the obtained samples were mechanically homogenized, and the supernatants were collected for analysis. The levels of MDA, GSH, SOD, and total iron in the brain tissues were determined using the MDA assay kit (Cat#BC0025, Solarbio), GSH assay kit (Cat#BC1175, Solarbio), SOD assay kit (Cat#BC0175, Solarbio), and iron assay kit (Cat#A039-2-1, njjcbio6), according to the manufacturer's protocols.

### Western Blot Analysis

Rat brain tissues comprising the infarcted area were collected and subjected to protein analysis by using the BCA protein detection kit (Beijing Soleibao Biotechnology Co., Ltd.) and an enzyme-labeling instrument (Infinite F50, TECAN). Approximately 30 μg of total protein was loaded into the SDS-PAGE gel and resolved at 120 V for 1 h. Thereafter, the protein components were transferred onto a PVDF membrane at 300 mA for 2 h. The membranes were blocked with 5% skimmed milk in PBST for 4 h at room temperature and incubated overnight with primary antibodies at 4°C. The contents were washed three times with PBST for 10 min each time. The membranes were incubated with secondary antibodies (goat anti-rabbit IgG) at 4°C for 4 h, treated with ECL solution, and then subjected to automatic chemiluminescence image analysis (Fine-do X6, Tanon). The following antibodies were used: anti-beta-Actin (1:10,000, D6A8, Abcam), anti-GPX4 (1:1,000, ab15251, Abcam), anti-FTH1 (1:500, ab27798, Abcam), anti-Tf (1:1,000, ab107166, Abcam), and anti-TfR1 (1:10,000, ab32197, Abcam). The stripe grayscale value was determined using ImageJ software.

### Real-Time Quantitative Polymerase Chain Reaction

Total RNA was isolated from the ischemic cerebral tissues by using the Trizol method (Life Technologies, United States) (17, 22). Thereafter, 1 μg of total RNA was reverse transcribed using a cDNA synthesis kit (N8050200, Life Technologies Holdings Pte Ltd.) according to the manufacturer's instructions, followed by real-time quantitative PCR (qRT-PCR) by using the SYBR kit. Primer sequences for the target genes, including that for β-actin, were included as an internal amplification control and are shown in the table below. Relative mRNA levels of the target genes were then analyzed.

The following primers were used:

GPX4-Forward primer: 5′-AATCCTGGCCTTCCCTTGCA-3′,
GPX4-Reverse primer: 5′-GCCCTTGGGCTGGACTTTCA-3′,
FTH1-Forward primer: 5′-CCAGAACTACCACCAGGACTC-3′,
FTH1-Reverse primer: 5′-GTTTCTCAGCATGTTCCCTCT-3′,
Tf-Forward primer: 5′-AAATGGAGATGGCAAAGAGG-3′,
Tf-Reverse primer: 5′-AGAGCCGAACAGTTGGAAGT-3′,
TfR1-Forward primer: 5′-ACTCTGCTTTGCGACTATTGC-3′,
TfR1-Reverse primer: 5′-TTCTGACTTGTCCGCCTCTT-3′,
β-Actin-Forward primer: 5′-CCCATCTATGAGGGTTACGC-3′,
β-Actin-Reverse primer: 5′-TTTAATGTCACGCACGATTTC-3′.

### Transmission Electron Microscope Analysis

Seven days after inducing MCAO, the left ventricles of 3 rats in each group were perfused with 0.9% sodium chloride solution after anesthesia. After waiting for the right side of the heart to bulge, we excised the right atrial ear until the effluent became colorless and then continued to inject 2.5% glutaraldehyde for fixation. The brain was quickly decapitated on ice for the separation of brain tissues, and ~1 × 1 × 1 mm brain volume was obtained as the ischemic area that was fixed in 2.5% glutaraldehyde solution for 12 h. The brain tissue samples were rinsed in PBS solution three times and fixed in 1% osmium acid solution for 2 h. Then, the samples were dehydrated in gradient ethanol, which was replaced with acetone, and then soaked and embedded in epoxy resin. After polymerization at −80°C for 24 h, the tissues were sliced into ~60–70-nm sections and subjected to 3% citrate-uranyl acetate double staining for observing changes in the mitochondrial structure of the ischemic tissue under a transmission electron microscope (TEM). Fragmentation and accumulation of the mitochondria around the nucleus were observed and quantified according to the method described by Anja et al. (23). Briefly, the cells containing a network of elongated mitochondria were classified as category I; cells containing fragmented but evenly distributed mitochondria were assigned to category II; and cells mainly containing fragmented mitochondria accumulated around the nucleus were classified as category III.

### Statistical Analysis

Data were analyzed with SPSS 23.0 (IBM, Armonk, NY, USA), and figures were prepared using Graph Pad Prism version 8.4.0 (GraphPad Software, San Diego, California, USA). Data are expressed as means ± SEM. One-way analysis of variance was used for comparisons across multiple samples, with significant differences between individual means analyzed using the Tukey's *post-hoc* test. *P*-values <0.05 were considered statistically significant, and *P*-values <0.01 were considered highly statistically significant.

## Results

### EA Treatment Improved Coordinated Motion Deficit in MCAO Rats

**Figure 1:**
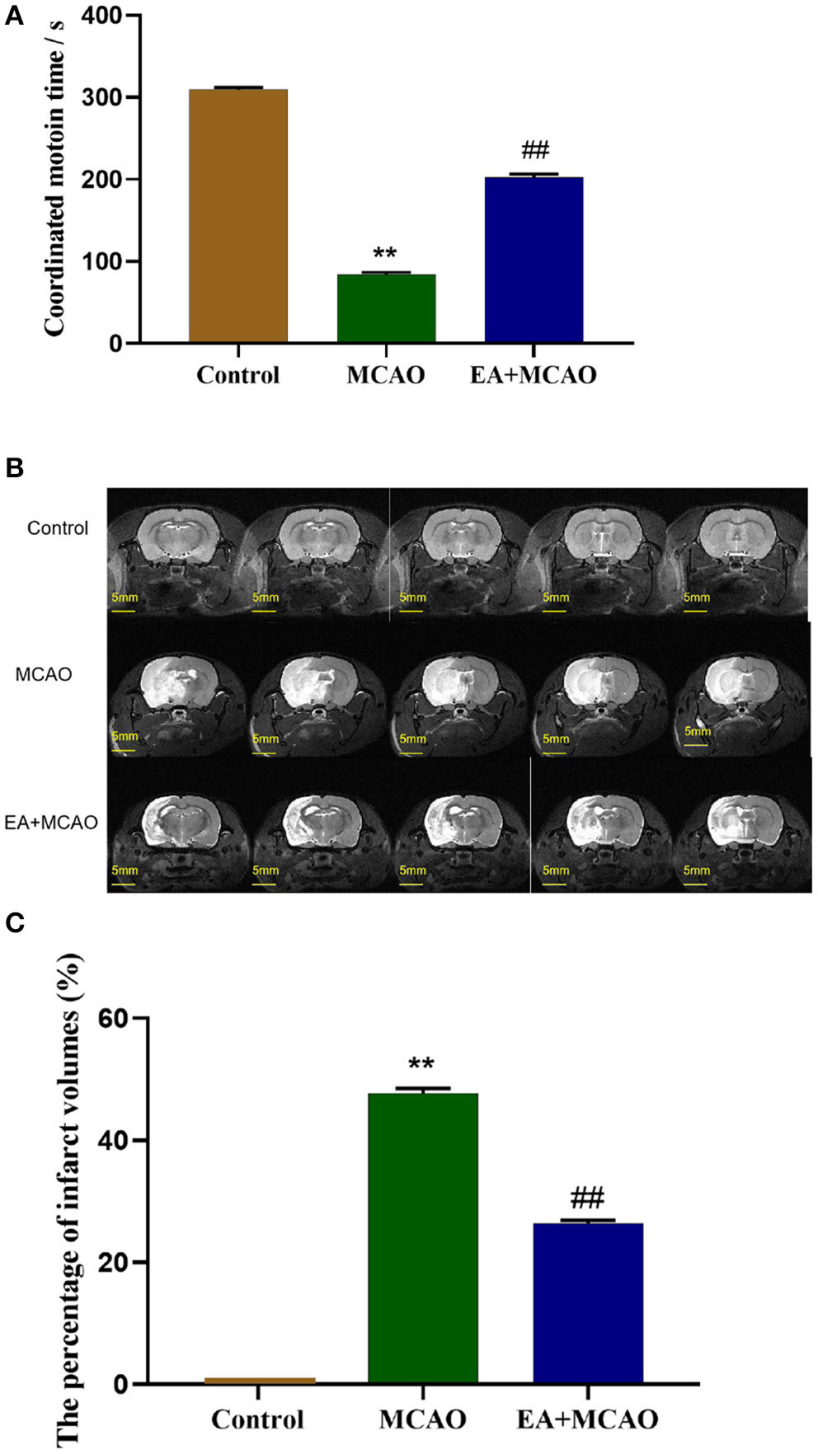
Coordinated motion time and cerebral infarction volume of rats in each group. **(A)** The coordinated motion time of rats in each group on the 7th day. **(B,C)** Evaluation of cerebral infarction volume of rats in different groups by 9.4T MRI T2-weighted imaging and analysis. Results are expressed as means ± SEM (*n* = 12, *n* = 3). **P < 0.01, compared with the Control group; ^##^*P* < 0.01, compared with the MCAO group.

The coordinated motion time was evaluated 7 days after surgery. As shown in Figure 1A, compared with the Control group, the rats in other groups displayed significantly decreased coordinated motion time (*P* < 0.01). However, compared with the MCAO group, EA significantly increased the coordinated motion time in the EA + MCAO group (*P* < 0.01).

### EA Treatment Reduces Infarct Volumes in MCAO Rats

The cerebral infarct volume was evaluated through T2-weighted imaging of 9.4T MRI on the 7th day after surgery. As shown in Figure 1B, the white area shown by the arrow is the site of cerebral infarction, and the gray area is the normal brain tissue. As shown in Figure 1C, the rats in the Control group showed no infarct volume. Compared with the Control group, large-scale cerebral infarction was noticed in the rats in the MCAO group (*P* < 0.01). However, EA decreased the cerebral infarction volume significantly in the EA + MCAO group compared with the MCAO group (*P* < 0.01).

### EA Treatment Inhibits Oxidative Stress and Reduces the Level of Iron in MCAO Rats

**Figure 2:**
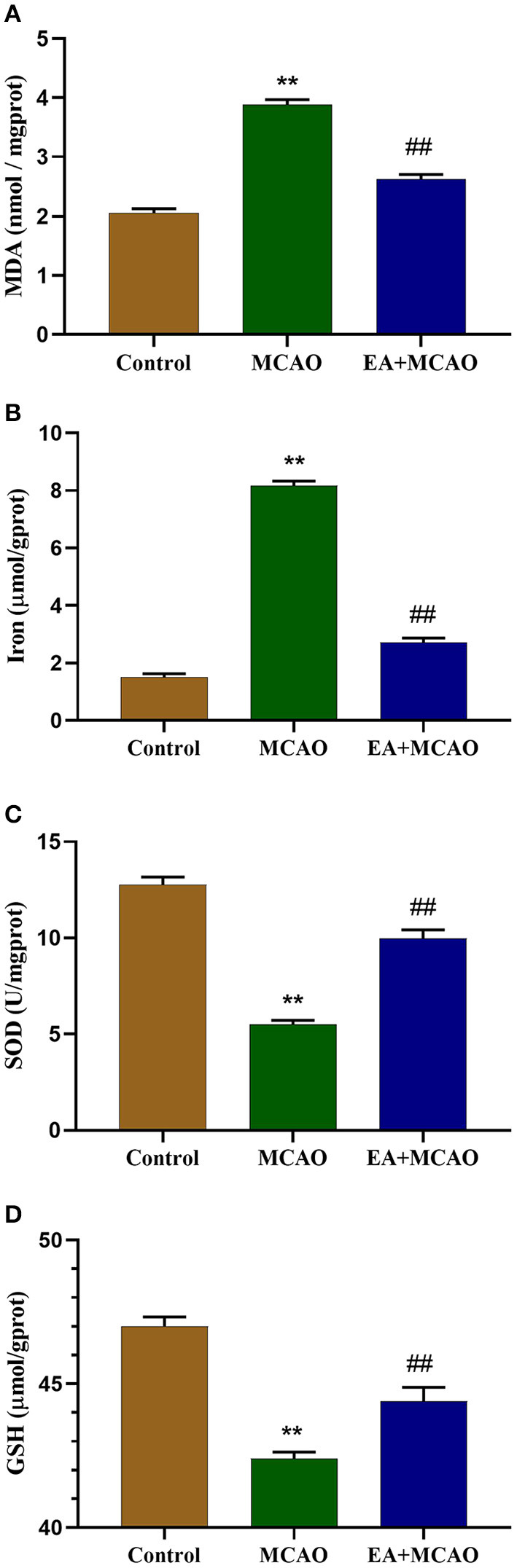
Levels of MDA **(A)**, total iron **(B)**, SOD **(C)**, and GSH **(D)** in the samples in each group on the 7th day. Results are expressed as means ± SEM (*n* = 6). **P < 0.01, compared with the Control group; ^##^*P* < 0.01, compared with the MCAO group.

As shown in Figures 2A,B, compared with the Control group, the levels of MDA and iron were found to be significantly increased in the MCAO group (*P* < 0.01 and *P* < 0.01, respectively). However, the levels of MDA and iron in the EA + MCAO group were significantly decreased compared with those in the MCAO group (*P* < 0.01 and *P* < 0.01, respectively). As shown in Figures 2C,D, compared with the Control group, the levels of SOD and GSH were significantly decreased in the MCAO group (*P* < 0.01 and *P* < 0.01, respectively); however, the levels of SOD and GSH were significantly increased in the EA + MCAO group compared with those in the MCAO group (*P* < 0.01 and *P* < 0.01, respectively).

### EA Treatment Regulates the Levels of Ferroptosis-Related Proteins

**Figure 3:**
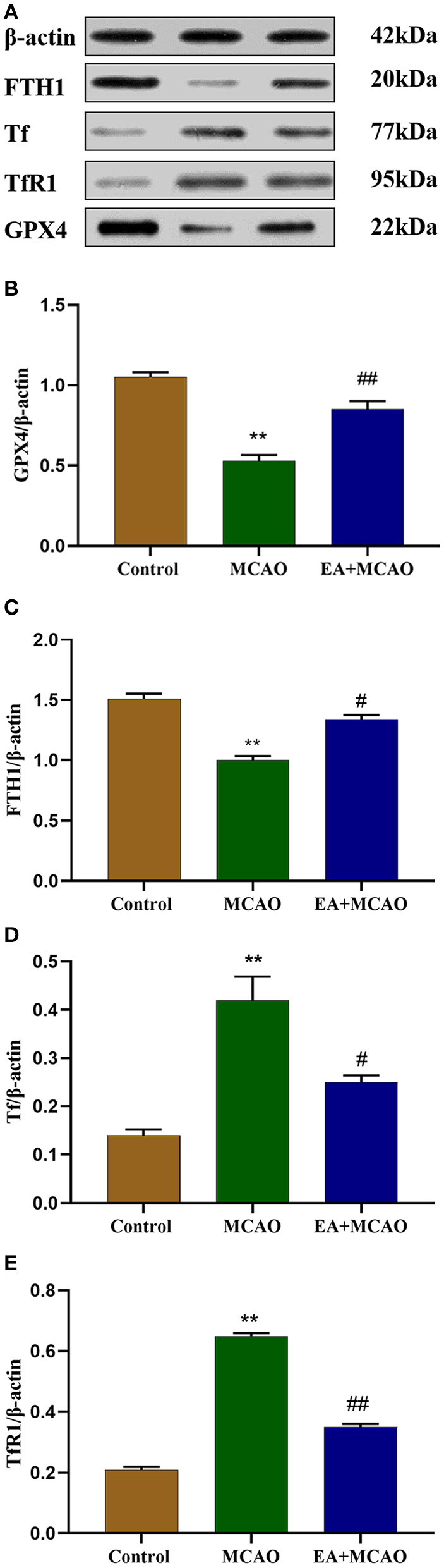
Protein bands **(A)** and levels of GPX4 **(B)**, FTH1 **(C)**, Tf **(D)**, and TfR1 **(E)** in each group. Results are expressed as means ± SEM (*n* = 6). **P < 0.01, compared with the Control group; ^#^*P* < 0.05 and ^##^*P* < 0.01, compared with the MCAO group.

Levels of ferroptosis-related proteins, namely, GPX4, FTH1, Tf, and TfR1, were determined through western blotting (WB) (Figure 3A). As shown in Figures 3B,C, the levels of GPX4 and FTH1 were significantly decreased in the MCAO group compared with those in the Control group (*P* < 0.01 and *P* < 0.01, respectively); however, EA intervention reversed this change in the EA + MCAO group (*P* < 0.01 and *P* < 0.05, respectively). As shown in Figures 3D,E, the levels of Tf and TfR1 were markedly increased in the MCAO group compared with those in the Control group (*P* < 0.01 and *P* < 0.01, respectively), but EA intervention decreased their levels in the EA + MCAO group compared with those in the MCAO group (*P* < 0.05 and *P* < 0.01, respectively).

### EA Treatment Regulates the mRNA Levels of Ferroptosis-Related Proteins

**Figure 4:**
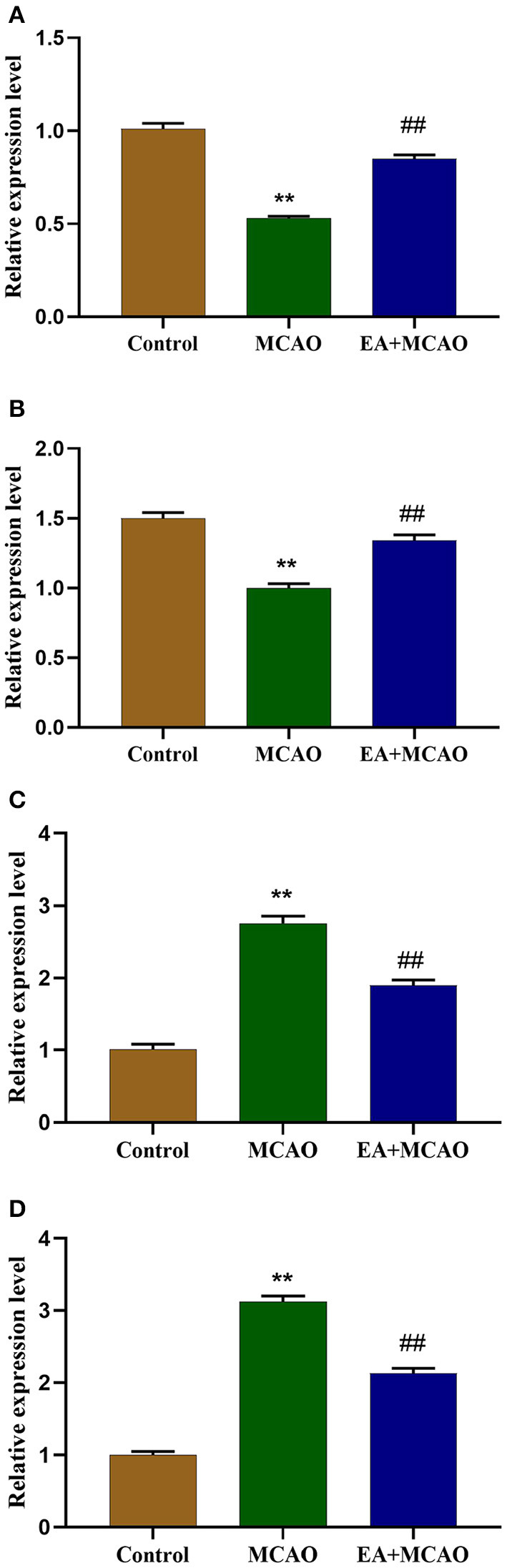
Levels of GPX4 mRNA **(A)**, FTH1 mRNA **(B)**, Tf mRNA **(C)**, and TfR1 mRNA **(D)** in each group. Results are expressed as means ± SEM (*n* = 6). **P < 0.01, compared with the Control group; ^##^*P* < 0.01, compared with the MCAO group.

The mRNA levels of ferroptosis-regulated proteins (GPX4, FTH1, Tf, TfR1) were determined through qRT-PCR. As shown in Figures 4A,B, compared with the Control group, the levels of GPX4 and FTH1 mRNA were markedly decreased in the MCAO group (*P* < 0.01 and *P* < 0.01, respectively), but EA intervention reversed this change in the EA + MCAO group compared with those in the MCAO group (*P* < 0.01 and *P* < 0.01, respectively). As shown in Figures 4C,D, compared with the Control group, the levels of Tf and TfR1 mRNA were significantly increased (*P* < 0.01, *P* < 0.01); however, EA decreased the level of Tf and TfR mRNA in the EA + MCAO group compared with those in the MCAO group (*P* < 0.01 and *P* < 0.01, respectively).

### EA Treatment Protects Neuronal Mitochondrial Injury in MCAO Rats

**Figure 5:**
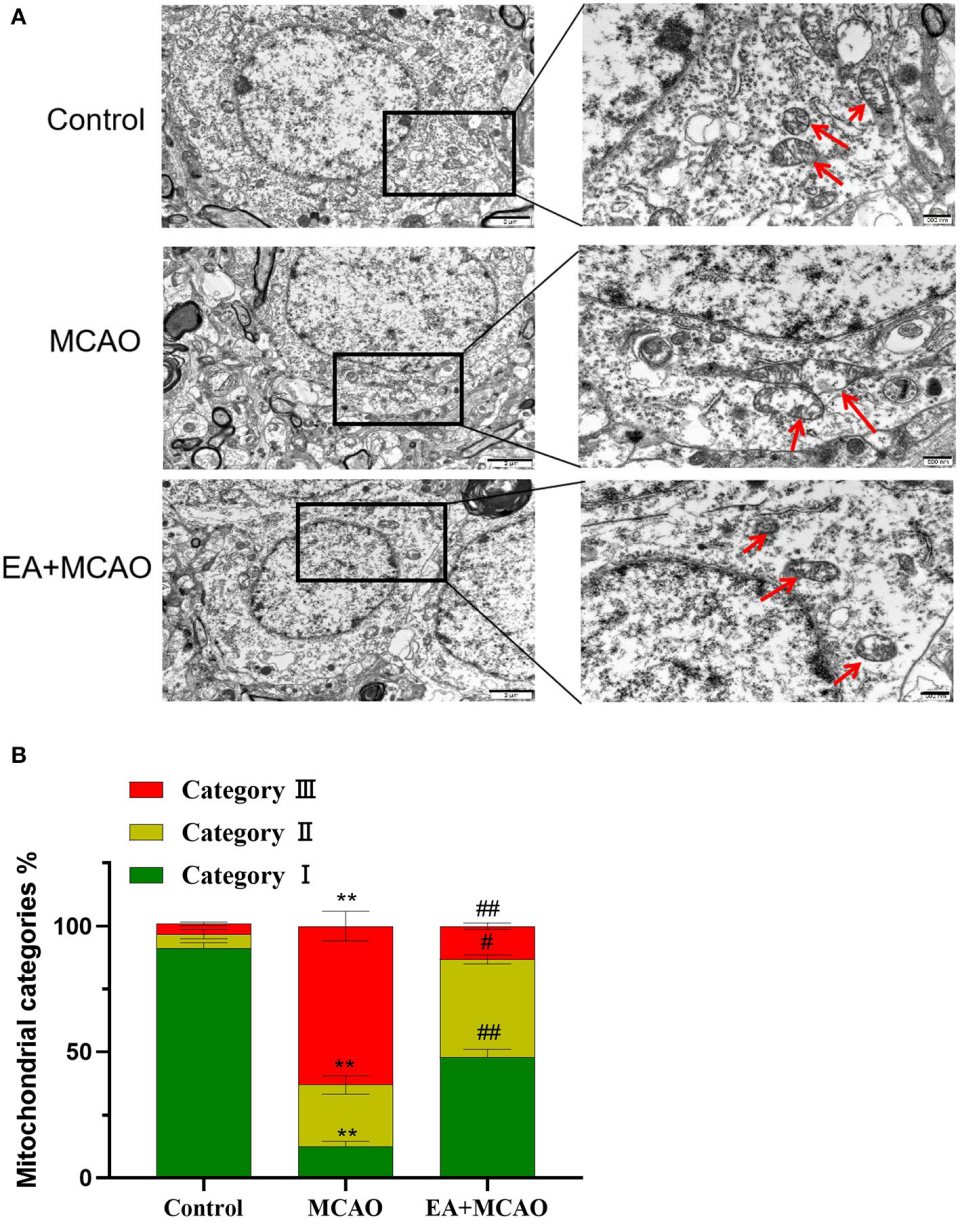
Electroacupuncture protects neuronal mitochondria. **(A)** TEM images of cortical cerebral tissues from each group. Scare bars: left, 2 μm; right, 500 nm. **(B)** Quantification of mitochondria counted blind to intervention of condition of three independent experiments. Results are expressed as means ± SEM. **P < 0.01, compared with the Control group; ^#^*P* < 0.05, and ^##^*P* < 0.01, compared with the MCAO group.

As shown in Figure 5A, the intracellular mitochondria were evenly distributed in a long reticular structure in the Control group, and their morphology was normal (outer membrane was complete and the crest was rich). In the MCAO group, many mitochondrial fragments, with a broken outer membrane and the decreased or disappeared crest, were found to be accumulated around the nucleus. In the EA + MCAO group, mitochondrial fragments were observed in the cytoplasm, but most of them were still uniformly distributed, and a few of them had accumulated around the nucleus. As shown in Figure 5B, compared with the Control group, the number of type I mitochondria decreased significantly (*P* < 0.01), whereas that of types II and III increased significantly (*P* < 0.01 and *P* < 0.01, respectively). Compared with the MCAO group, the number of type I mitochondria increased significantly (*P* < 0.01), whereas that of types II and III decreased significantly (*P* < 0.05 and *P* < 0.01, respectively).

## Discussion

Ischemic stroke is caused by a sudden interruption of blood flow to the brain, resulting in brain cell death and neurological dysfunction (24). With increase in the aging population, the number of patients with stroke is also increasing and is predicted to reach 77 million by 2030 (25). However, only a few effective thrombolytic drugs have been developed and the time period for immediate treatment is very short. EA is one of the common treatment methods in clinical practice that offers advantages of adjustable strength, frequency, and easy quantification (26). In this study, we selected the main acupuncture point of the “Awakening and Opening the Brain” method developed by an academician, Shi Xuemin, of Tianjin University of Chinese Medicine. Clinical studies have shown that this method can considerably improve the neurological function, brain metabolism, and quality of life of patients (27, 28).

In 2012, Dixon et al. discovered and proposed ferroptosis, which emphasizes the iron-dependent cell death caused by the accumulation of lipid peroxides and related metabolites and mass consumption of polyunsaturated fatty acids in the plasma membrane (29). Morphologically, ferroptosis causes condensation of the intracellular mitochondrial membrane, rupturing of the outer membrane, and reduction or disappearance of the crest formed in the inner membrane (30). In terms of biochemical changes, GPX4 and SOD activities decrease, GSH is consumed in large quantities, and intracellular lipids are oxidized by ferrous ions through chemical reactions similar to Fenton, generating a large number of metabolites (MDA and 4-HNE) and active free radicals (ROS, RNS, and RLS) (31, 32). GPX4 can reduce harmful active free radicals into harmless alcohols, and GSH is the major neurotransmitter and endogenous antioxidant in the brain (33). Given that the GPX4–GSH–cysteine axis is the central node of the ferroptotic death cascade, RSL3 (RAS selective lethal 3, a ferroptosis inducer) can act on GPX4 to induce ferroptosis in cells (34).

Moreover, ferric homeostasis proteins levels were altered. Under physiological conditions, iron in the plasma is closely bound to transferrin and enters into cells by means of endocytosis through transferrin receptors found on the cell membrane surface to form endosomes. In an acidic environment, iron ions are released from transferrin, catalyzed by ferrous reductase to form ferrous ions, and finally transported to the cytoplasm by divalent transporters (DMT) on the endoplasmic surface to perform physiological functions (35). When the iron content is high, it is stored in the form of ferritin, a heterogeneous polymer composed of ferritin light chains (FLH) and ferritin heavy chains (FTH) that releases iron for cellular use at low levels through NCOA4-mediated selective ferritin autophagy (36). When ferroptosis occurs, the expression of transferrin and transferrin receptor increases; TFR1 has been shown to be a specific marker of ferroptosis, which increases the absorption of iron by cells (37, 38), On the other hand, NCOA4-mediated ferritin autophagy also increases (36).

In this study, we determined the ferroptosis-inhibiting effects of EA in MCAO rats. Our results indicated that EA can significantly increase the activity of GPX4 and SOD and the level of GSH and reduce the accumulation of MDA and iron. We verified the effect of EA on antioxidant stress, and our result is consistent with those of Lin et al. (16). In addition, EA protects the mitochondria in the ischemic brain tissue. To further determine the regulatory effect of EA on iron homeostasis-related proteins, WB and RT-qPCR were used to detect the levels of FTH1, Tf, and TfR1. We found that EA can increase the level of FTH1 and decrease the levels of Tf and TfR1. These results indicate that EA alleviates ICS by inhibiting ferroptosis. However, our study has some limitations. Although ferroptosis involves many processes and mechanisms, we focused only on changes in the oxidative stress level, iron homeostasis-related proteins, and mitochondrial structure in this study. Moreover, the pathways or mechanisms through which EA inhibits ferroptosis and regulates iron homeostasis remain unclear. In this study, we used only the MCAO rat model; although this model is often used in the ischemic stroke studies and displays high similarity to that of the human cerebral infarct model, it may not be an absolute representative. The results of this study require further validation using other animals and *in vitro* cell models.

## Data Availability Statement

The raw data supporting the conclusions of this article will be made available by the authors, without undue reservation.

## Ethics Statement

The animal study was reviewed and approved by Experimental Animal Ethics Committee of Anhui University of Chinese Medicine.

## Author Contributions

YH designed the experiments. GL performed the experiments and wrote the article. JD performed the experiments and analyzed the data. XL obtained the data. All authors contributed to the article and approved the submitted version.

## Conflict of Interest

The authors declare that the research was conducted in the absence of any commercial or financial relationships that could be construed as a potential conflict of interest.
